# Radiation oncology resident training in patient safety and quality improvement: a national survey of residency program directors

**DOI:** 10.1186/s13014-018-1128-5

**Published:** 2018-09-24

**Authors:** Matthew B. Spraker, Matthew J. Nyflot, Kristi R. G. Hendrickson, Stephanie Terezakis, Shannon E. Fogh, Gabrielle M. Kane, Eric C. Ford, Jing Zeng

**Affiliations:** 1 Department of Radiation Oncology, Washington University in St. Louis, 4921 Parkview Place, CAM LL, CB 8224, St. Louis, MO 63110 USA; 2 Department of Radiation Oncology, University of Washington, Seattle, WA USA; 3 Department of Radiation Oncology and Molecular Radiation Sciences, Johns Hopkins University, Baltimore, MD USA; 4 Department of Radiation Oncology, University of California San Francisco, San Francisco, CA USA

**Keywords:** Safety, Quality improvement, Education, Residency

## Abstract

**Background:**

Physicians and physicists are expected to contribute to patient safety and quality improvement (QI) in Radiation Oncology (RO), but prior studies suggest that training for this may be inadequate. RO and medical physics (MP) program directors (PDs) were surveyed to better understand the current patient safety/QI training in their residency programs.

**Methods:**

PDs were surveyed via email in January 2017. Survey questions inquired about current training, curriculum elements, and barriers to development and/or improvement of safety and QI training.

**Results:**

Eighty-nine RO PDs and 84 MP PDs were surveyed, and 21 RO PDs (28%) and 31 MP PDs (37%) responded. Both RO and MP PDs had favorable opinions of current safety and QI training, and used a range of resources for program development, especially safety and QI publications. Various curriculum elements were reported. Curriculum elements used by RO and MP PDs were similar, except RO were more likely than MP PDs to implement morbidity and mortality (M&M) conference (72% vs. 45%, *p* < 0.05). RO and MP PDs similarly cited various barriers, but RO PDs were more likely to cite lack of experience than MP PDs (40% vs. 16%, *p* < 0.05). PDs responded similarly independent of whether they reported using a departmental incident learning system (ILS) or not.

**Conclusions:**

PDs view patient safety/QI as an important part of resident education. Most PDs agreed that residents are adequately exposed to patient safety/QI and prepared to meet the patient safety/QI expectations of clinical practice. This conflicts with other independent studies that indicate a majority of residents feel their patient safety/QI training is inadequate and lacks formal exposure to QI tools.

**Electronic supplementary material:**

The online version of this article (10.1186/s13014-018-1128-5) contains supplementary material, which is available to authorized users.

## Background

Patient safety/quality improvement (QI) is recognized as an essential part of radiation oncology (RO) practice. Physicians and physicists are expected to contribute to patient safety/QI [1], and will be responsible for ensuring the safety of increasingly sophisticated and complex treatments [2] delivered in an environment where quality indicators impact reimbursement [3]. The Accreditation Council for Graduate Medical Education (ACGME) has identified core learning objectives focused on patient safety/QI for inclusion in RO resident training [4]. It is therefore surprising that only a minority of RO and medical physics (MP) residents reported that their training in patient safety/QI concepts is adequate [5]. Most are unfamiliar with important concepts such as root cause analysis (RCA) [6] and are unsatisfied with their patient safety/QI training [7].

Unfortunately, the process and challenges associated with creating and improving resident safety and QI training remain elusive in RO. This study conducted a national survey of PDs of RO and MP residency programs to better understand the current training, curriculum elements, and barriers to development of patient safety/QI training.

## Methods

PDs of RO residency programs accredited by the ACGME and therapeutic MP programs accredited by the Commission on Accreditation of Medical Physics Education Programs were identified. The survey was distributed via email using REDCap electronic data capture tools hosted at our institution [8]. Responses were anonymous. The study was deemed exempt from review by the local Institutional Review Board.

The survey included questions assessing demographics, state of current safety/QI training, resources used in development and/or improvement of training, curriculum elements used, and how PDs perceived and assessed the effectiveness of training. The survey was developed by the authors and pilot tested within an academic RO department prior to release (see Additional file 1: for complete survey).

Responses to questions that used the Likert scale were collapsed to a three-point scale for analysis (i.e. “strongly disagree”/“disagree” and “agree”/“strongly agree” combined). Previous research suggests that resident safety and QI training experience may be related to the presence of departmental RO incident learning system (ILS) [5], so responses were compared for participants that reported having a departmental lLS versus those that did not. The differences were tested for statistical significance in Microsoft Excel (v14.6.5) using chi-square tests for Likert scale questions and unpaired t-tests otherwise. *P* < 0.05 was considered significant.

## Results

### Participant characteristics

Eighty-nine RO PDs and 84 MP PDs were surveyed, and 21 RO PDs (28%) and 31 MP PDs (37%) responded. Three responses were excluded for failure to identify whether they were RO or MP PDs. RO programs ranged in size from 10 or less residents (*N* = 18), 11–16 residents (*N* = 6), and more than 16 residents (N = 1). MP programs had 2 or less (*N* = 16) or more than 2 residents (*N* = 14). Most participants (61%) reported that their department is using a RO specific ILS and 58% reported that faculty in their departments present or publish patient safety/QI research.

### RO and MP PD’s opinions of patient safety/QI training

**Fig. 1:**
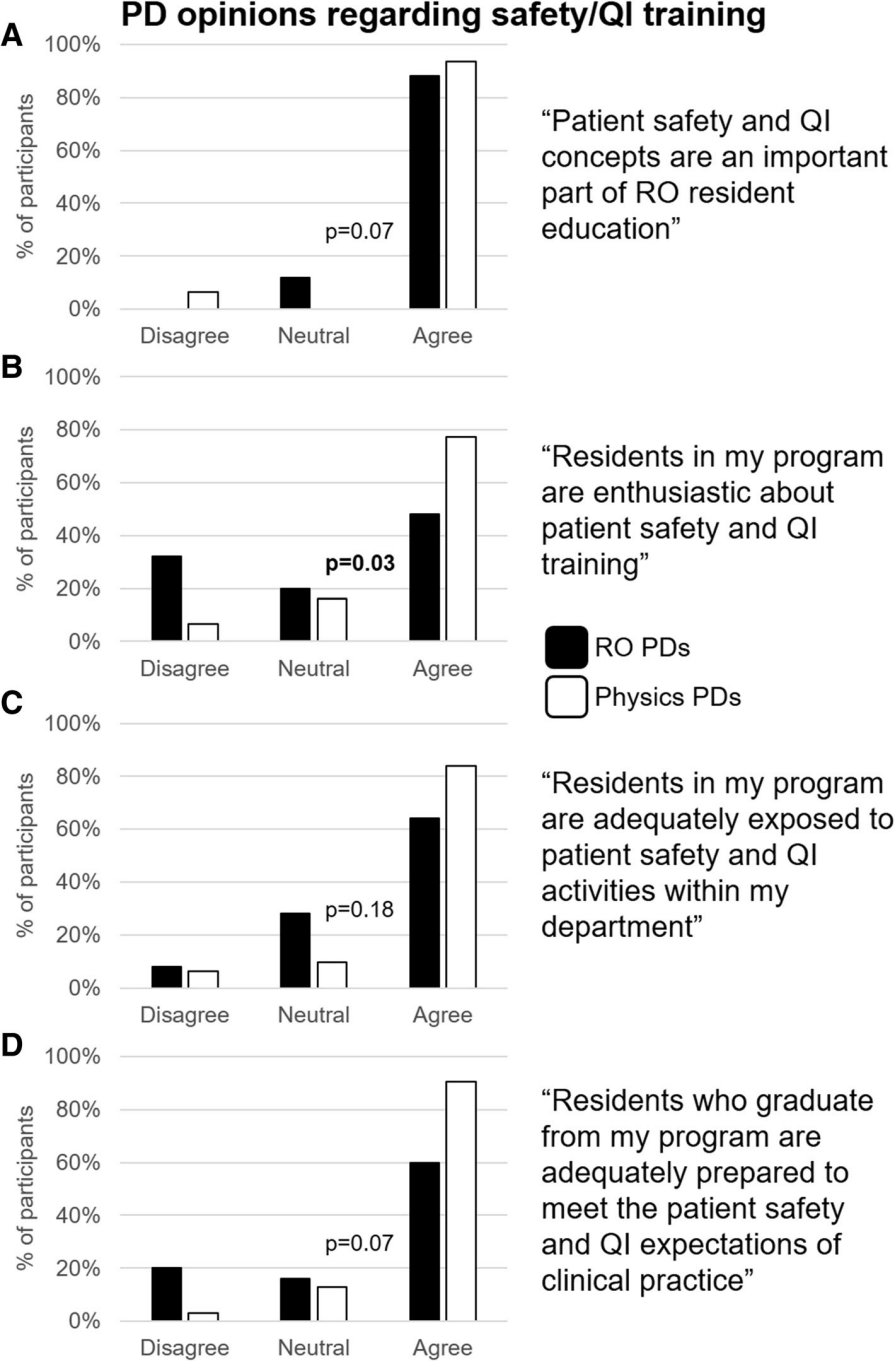
**a-d.** Depicts PD opinions regarding residents and their patient safety and QI training. MP PDs (white) more commonly agreed with all statements than RO PDs (black), but only the difference in responses for about residents’ enthusiasm (**b**) reached significance (*p* = 0.03). RO = Radiation Oncology, MP = Medical Physics, PD = Program Director, QI = Quality Improvement

PD opinions of resident safety and QI training are shown in Fig. 1a-d. The vast majority of RO and MP PDs (88% vs. 94%) reported that patient safety and QI are an important part of resident education. MP PDs were more likely than RO PDs to agree that residents in their program were enthusiastic about safety training (77% vs. 48%, Fig. 1b). Otherwise, MP and RO PDs responses were similar.

### Resource utilization in creating and/or improving safety and QI training programs

**Fig. 2:**
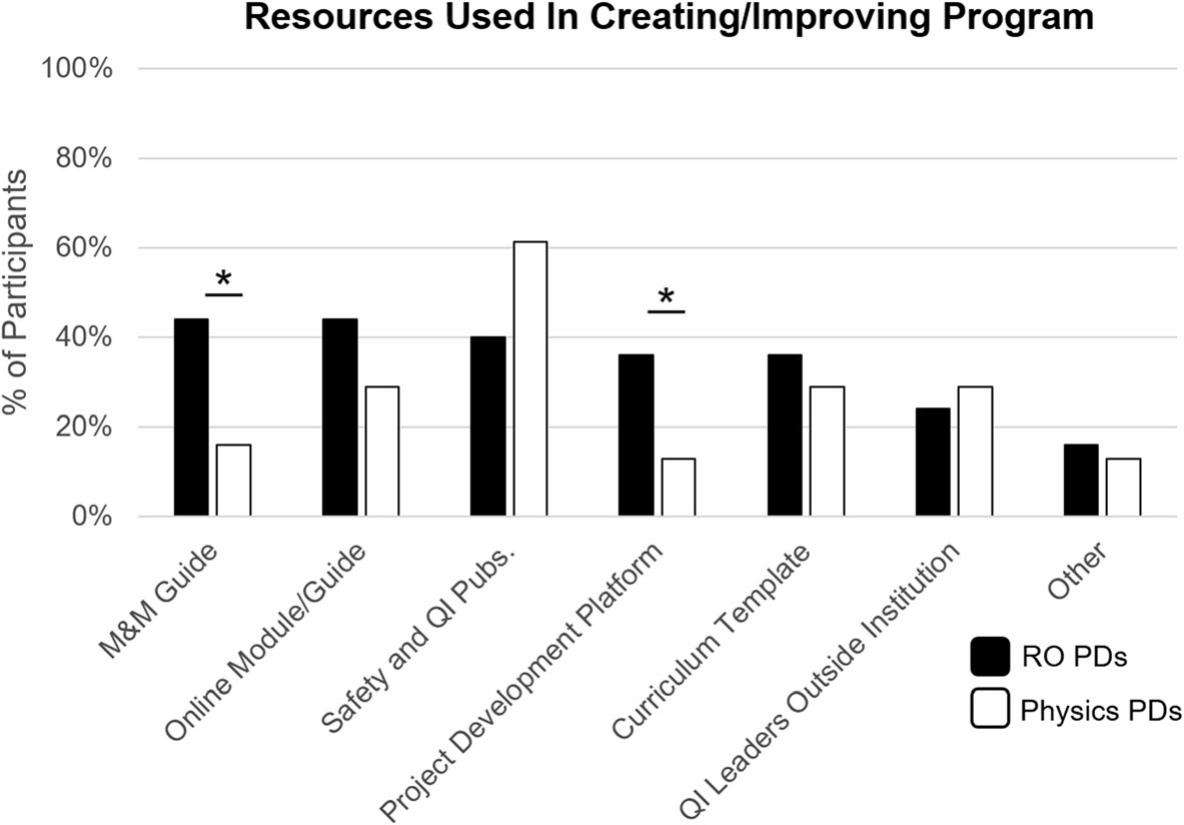
Depicts the resources used by RO (black) and MP PDs (white) in creating their resident patient safety and QI training experience. RO PDs were significantly more likely than MP PDs to use an M&M facilitator guide and a project development platform (both *p* < 0.05). RO = Radiation Oncology, MP = Medical Physics, PD = Program Director, M&M = Morbidity and Mortality, Pubs. = Publications

The person primarily responsible for developing and implementing the patient safety/QI training experience was most commonly the residency PD (38%), but other faculty members (25%) or the medical director (21%) also lead these efforts. RO and MP PDs reported using a range of resources, including patient safety/QI publications (44% vs. 58%, *p* = 0.68) (Fig. 2). More RO than MP PDs reported using a morbidity and mortality (M&M) guide (48% vs. 13%, *p* < 0.005) and a QI project development tool (36% vs. 13%, *p* < 0.05), such as Plan-Do-Study-Act (PDSA). Otherwise, a range of resources were used similarly (Fig. 2).

### Curricular elements and assessing effectiveness of patient safety/QI training programs

**Fig. 3:**
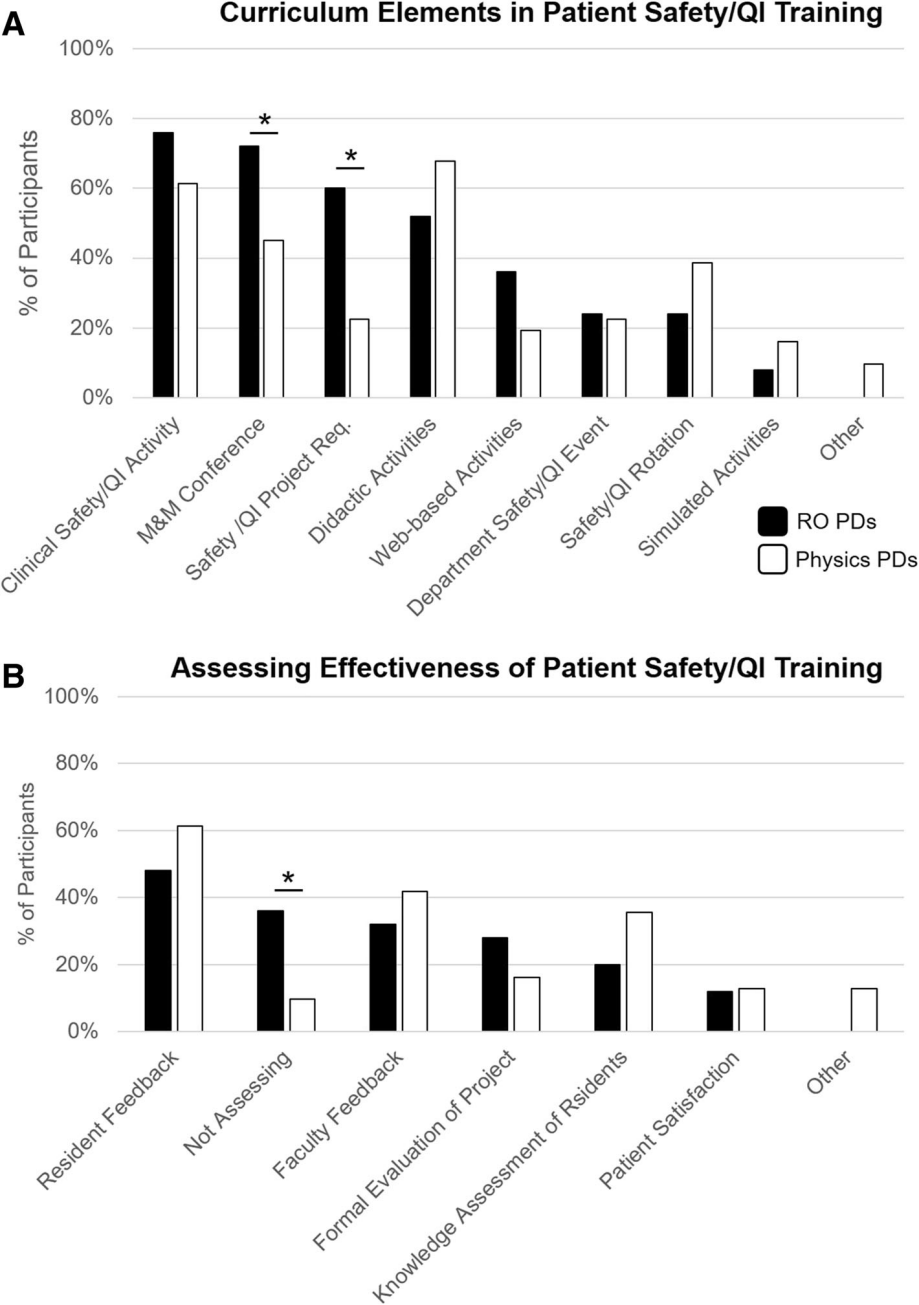
**a.** Depicts the curricular elements utilized by RO (black) and MP (white) PDs in their resident patient safety and QI training programs. A clinical safety and QI activity, such as a root cause analysis, was commonly used by both RO and MP PDs. RO PDs reported using M&M conference and a safety/QI project requirement more often than MP PDs. **b**. Depicts how RO (black) and MP (white) PDs are assessing the effectiveness of their patient safety and QI training program. *p < 0.05. RO = Radiation Oncology, MP = Medical Physics

A range of curriculum elements were similarly implemented by RO and MP PDs in resident patient safety/QI training programs (Fig. 3a). These included a safety/QI activity (i.e. RCA), M&M conference, safety/QI project, and didactic activities (Fig. 3), which were all used by greater than 50% of PDs. The only significant differences between RO and MP PDs were that RO PDs reported using safety/QI project requirements for graduation (60% vs. 23%, *p* < 0.005) and M&M conference as required learning (72% vs. 45%, *p* < 0.05) more often than MP PDs. Four RO and 5 MP participants provided detailed information about a dedicated patient safety/QI rotation. The safety rotations range from 3 weeks to 6 months in duration, and residents had 10–100% of their time directed toward patient safety/QI education on the rotation.

Both RO and MP PDs assessed the effectiveness of their programs using similar methods, most commonly resident feedback (RO 48% vs. MP 61%, *p* = 0.33) and faculty feedback (RO 32% vs. MP 42%, *p* = 0.45) (Fig. 3b). RO were more likely than MP PDs to report that they are not assessing the effectiveness of their safety training program (36% vs. 10%, *p* < 0.05).

### Barriers to creating and/or improving patient safety/QI training programs

**Fig. 4:**
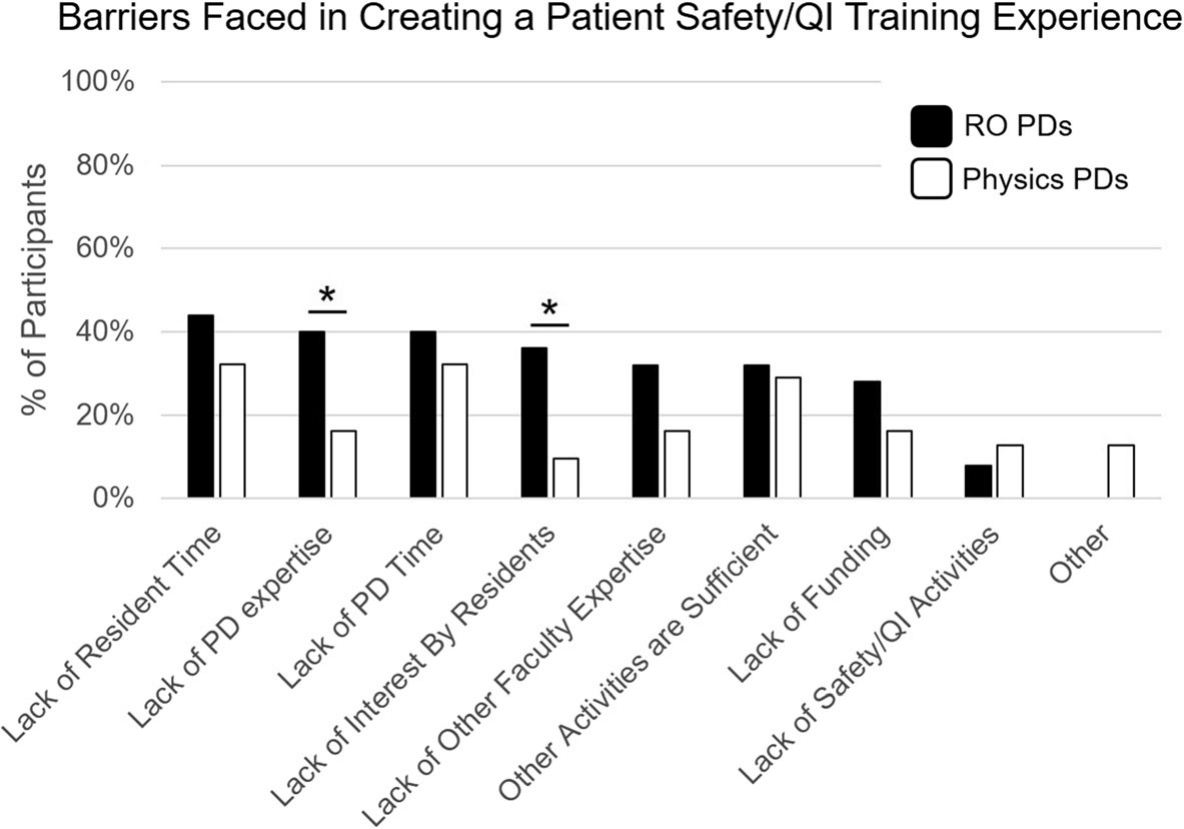
Depicts barriers faced by RO (black) and MP (white) PDs in creating a resident patient safety and QI training experience. RO were more likely than MP PDs to report lack of PD expertise and lack of interest by residents as barriers (both p < 0.05). RO = Radiation Oncology, MP = Medical Physics

PDs reported a range of barriers faced in creating and improving a patient safety/QI training experience, but each was reported at a relatively low rate (Fig. 4). Responses were similar for RO and MP PDs, except RO PDs were more likely to report lack of expertise as a barrier to creating or improving their training programs (40% vs. 16%, *p* < 0.05). Ninety-six percent of participants reported that safety culture was not a barrier, and most reported RO and MP PDs reported that they had adequate access to patient safety/QI leaders in their institution (88% vs. 77%, *p* = 0.22).

Seventeen participants offered free-text responses to the question “What advice would you have for program directors seeking to improve the patient safety/QI resident training experience in their program?” These are shown in the Additional file 1. Six described the importance of integrating residents into institutional patient safety/QI activities, and 6 noted the importance of institutional support and culture for improving training.

### The influence of departmental use of incident learning systems

Additional file 1 Tables S1 and S2 depict the responses from PDs reporting use of a departmental ILS versus those who did not. PDs with a departmental ILS were less likely to implement a patient safety/QI educational event (e.g. safety research day) than those without a departmental ILS (14.7% vs. 36.8%, *p* = 0.05). Otherwise there were no significant differences between groups.

## Discussion

The goals of this national survey were to understand the current status of patient safety/QI training experiences and identify challenges faced by PDs in developing patient safety/QI training programs. The survey identified important differences between RO and MP PDs and between PDs and prior surveys of residents. These findings may guide improvement of resident patient safety/QI training.

### Differences between PD and resident opinions of patient safety/QI training

**Fig. 5:**
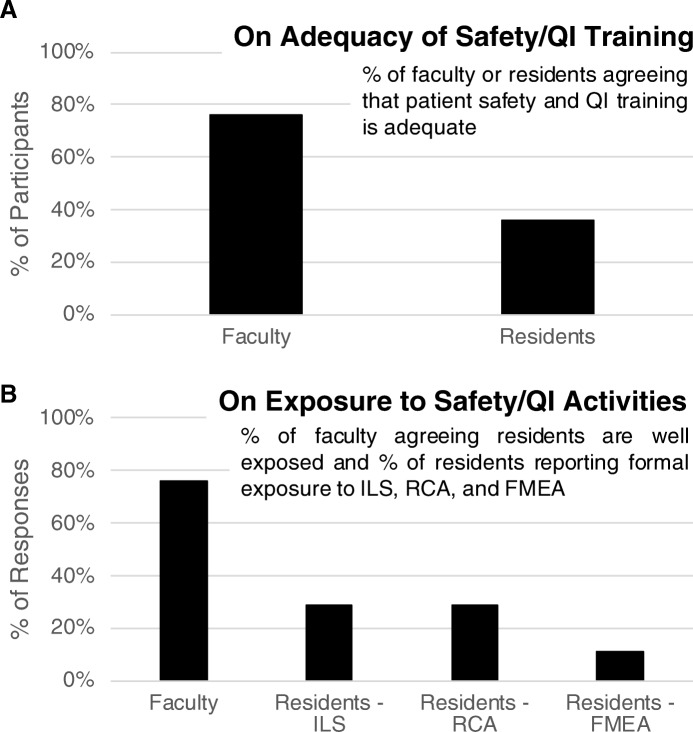
**a**. Depicts responses from faculty and residents regarding the adequacy of resident patient safety and QI training. Faculty = % agreeing with the statement: “I feel that residents who graduate from my program are adequately prepared to meet the patient safety and QI expectations of clinical practice”. Residents = % agreeing with the statement: “I feel that the formal teaching of principles in patient safety and quality management is adequate in my radiation oncology residency”. **b**. Depicts responses from faculty and residents regarding exposure to patient safety and QI activities during training. Faculty = % agreeing with the statement: “I feel that residents who graduate from my program are adequately prepared to meet the patient safety and QI expectations of clinical practice.”. Residents = % reporting “formal exposure” or “practical experience” with ILS, RCA, and FMEA. ILS = Incident learning system, RCA = root cause analysis, FMEA = failure modes and effects analysis. Note: resident responses are adapted from Spraker et al. [5]

Most PDs agreed that residents are adequately exposed to patient safety/QI activities (64% RO vs. 84% MP) and are adequately prepared to meet these expectations of clinical practice (60% MD vs. 90% MP). This conflicts with a recent survey of RO and MP residents [5], which shows that less than 40% feel their patient safety/QI training is adequate and most lack formal exposure to key concepts, such as ILS reporting, RCA, and failure modes and effects analysis (FMEA) (Fig. 5a-b), although the survey questions are not identical. A prior survey found low RO and MP resident satisfaction with patient safety/QI training [7] and a recent study by the ACGME also found that most residents have a limited knowledge of RCA [6].

### Differences between RO and MP PD opinions of resident safety and QI training

MP PDs had more favorable opinions of resident safety and QI training than RO PDs, with differences in the question about resident enthusiasm reaching statistical significance. This might reflect differences in the clinical expectations and training goals between physicians and physicists. Physicists clinical duties have substantial overlap with safety and QI activities, and they accept primary responsibility of the clinical safety and QI program alongside physicians [9–11]. This may make practicing physicists especially well-equipped to teach certain aspects of patient safety/QI, such as RCA and FMEA. The finding that MP PDs were less likely than RO PDs to cite lack of expertise as a barrier to program development supports this hypothesis. This also may explain why MP residents are more likely than RO residents to be well-exposed and adequately trained in FMEA [5].

A recent international Delphi study recommended patient safety/QI competencies for RO residents that require proficiency in both procedural and non-technical areas, such as how to engage patients to champion the safety and quality of their treatment [12]. RO educators’ scope of practice including non-technical aspects of patient safety/QI compared to MP PDs may explain why they are more likely to use a M&M facilitator guide and implement M&M.

### The current state and future directions of resident patient safety/QI training

Participants most frequently reported using publications and online modules or guides as resources to create and/or improve patient safety/QI training programs. These resources are becoming increasingly available. They include a detailed list of patient safety/QI competencies for RO residents [12], a framework for didactics [13] and web-based modules (i.treatsafely.org, IAEA e-Learning course (www.iaea.org/resources/rpop), Institute for Healthcare Improvement (ihi.org), and a safety culture education program [14]. Additionally, a patient safety education program including a dedicated safety and QI rotation designed for MP residents has been published [15].

We propose two recommendations for improvement of resident patient safety/QI training. First, we recommend a more formalized collaboration between MP and RO PDs or inclusion of patient safety/QI champions to close the gap of self-reported lack of expertise in these topics as a barrier to effective training. Institutional support through faculty development would further augment RO and MP collaboration. Second, PDs should consider integrating resident training into institutional patient safety/QI activities. This was recommended in the free text responses of six participants, is consistent with recommendations from the ACGME [16], and offers exposure to more faculty with expertise in patient safety and QI when this might be lacking in smaller RO programs. Building safety and QI training programs around the departmental ILS may be another effective approach as it enhances effective situated learning [15], and has been linked to an improved patient safety/QI training experience [5].

This study is limited by a response rate of only 28% and 37% for RO and MP PDs, respectively. This is similar to other surveys in this field where response rates range from 10 to 33% [5, 14, 17–19]. The responses may not represent the opinions of all PDs. Indeed, over 60% of participants reported using a departmental ILS and half reported publishing or presenting patient safety/QI research. This group may have more experience and exposure to patient safety/QI than average, and this could have biased their responses toward more favorable opinions of patient safety/QI training and toward reporting less barriers and challenges than other PDs face. Another limitation is that the findings reaching statistical significance were not corrected for multiple comparisons. However, the goal of this paper was to identify the current state of patient safety/QI education from the perspective of PDs, not rigorously compare responses between groups.

## Conclusions

This national survey of RO and MP PDs found that PDs view patient safety and QI as an important part of resident education. PDs use a range of available resources to implement diverse educational activities. Participants identified several important barriers to creating and improving patient safety/QI training, including lack of expertise. Most PDs agreed that residents are adequately exposed to patient safety/QI and prepared to meet the patient safety/QI expectations of clinical practice. This conflicts with other independent studies that indicate a majority of residents feel their patient safety/QI training is inadequate and lacks formal exposure to QI tools.

## Additional file

Additional file 1: **Figure S1.** “What advice would you have for program directors seeking to improve the patient safety and QI resident training experience in their program?”. Depicts free text responses of the survey participants, edited for brevity and clarity. **Table S1.** Program directors’ (PDs) responses regarding residents and their patient safety and QI training by Incident Learning System (ILS) use. Depicts PD responses split by those reporting departmental use of an ILS versus those not reporting ILS use (No ILS). *P*-values are reported for the chi-square test. RO = Radiation Oncology, QI = Quality Improvement. **Table S2.** Program directors’ (PDs) reporting of resources used, curricular elements used, how effectiveness is assessed, and barriers faced in creating and/or improving patient safety and QI programs by Incident Learning System (ILS) use. Responses are split by those reporting departmental use of an ILS versus those not reporting ILS use (No ILS). P-values are reported for t-tests between groups. RO = Radiation Oncology, QI = Quality Improvement, M&M = Morbidity and Mortality, PD = Program Director. (DOCX 27 kb)

## Abbreviations

ACGME: Accreditation Council for Graduate Medical Education
ASTRO: American Society for Radiation Oncology
FMEA: Failure Modes and Effects Analysis
ILS: Incident Learning System
MP: Medical Physics
PD: Program Director
QI: Quality Improvement
RCA: Root Cause Analysis
RO: Radiation Oncology
