# Supplementary material for: Radiation oncology resident training in patient safety and quality improvement: a national survey of residency program directors
Source: Radiat Oncol. 2018 Sep 24;13:186. doi: 10.1186/s13014-018-1128-5 (PMC6154943; doi:10.1186/s13014-018-1128-5)
Supplement: Supplementary file 1 — Figure S1. “What advice would you have for program directors seeking to improve the patient safety and QI resident training experience in their program?”. Depicts free text responses of the survey participants, edited for brevity and clarity. Table S1. Program directors’ (PDs) responses regarding residents and their patient safety and QI training by Incident Learning System (ILS) use. Depicts PD responses split by those reporting departmental use of an ILS versus those not reporting ILS use (No ILS). P-values are reported for the chi-square test. RO = Radiation Oncology, QI = Quality Improvement. Table S2. Program directors’ (PDs) reporting of resources used, curricular elements used, how effectiveness is assessed, and barriers faced in creating and/or improving patient safety and QI programs by Incident Learning System (ILS) use. Responses are split by those reporting departmental use of an ILS versus those not reporting ILS use (No ILS). P-values are reported for t-tests between groups. RO = Radiation Oncology, QI = Quality Improvement, M&M = Morbidity and Mortality, PD = Program Director. (DOCX 27 kb) [file 13014_2018_1128_MOESM1_ESM.docx]

**“What advice would you have for program directors seeking to improve the patient safety and QI resident training experience in their program?”**

**(Edited for brevity and clarity)**

***Comments Describing Integrating Resident Education into Institutional Activities***

1. “Engage residents from a multi-dimensional approach. Building a strong culture of safety/quality helps facilitate educational goals.”
2. “I don't think online or classroom lectures are useful for these topics. Hands on experience/workshops of practical applications would be a much better way to teach and implement these principles.”
3. “Keep talking about it in your research meetings. Encourage QI projects to be submitted within the institution. Encourage residents to submit their work to ACGME patient safety meetings.”
4. “Involve residents directly in continuous quality improvement meetings”
5. “Have residents participate in departmental quality assurance and quality control meetings.”
6. “Make it part of the curriculum and make sure senior residents attend quality assurance and safety meetings. Also, an annual safety refresher is good.”

***Comments Describing the Importance of Institutional Support and Culture***

1. “Ensure the department overall has a dedication to patient safety. Find a physics faculty with a passion for patient safety.”
2. “Get administrative-level buy in. A departmental incident learning system is a key to making it easy to create the curriculum around it and that data.”
3. “Have a department champion and invest in their education.”
4. “Advocate for funding and time to direct and evaluate these activities.”
5. “Emphasize to the administration the critical role of safety practices in radiation oncology. The departmental leadership needs to ascertain the importance of the provision and acquisition of commensurate resources for these activities.”
6. “Online patient safety/QI modules are just now being developed. Stay tuned for info on this from several institutions.”

***Other Comments***

1. “It is a work in progress. Start small and build on it. I wish we were further along, but there is a lot of material already packed into a 2-year program. It is challenging to add a safety component without compromising elsewhere.”
2. “Talk to other programs.”
3. “Learn about quality improvement. Get outside help”
4. “Set up clear, reasonable and achievable expectations.”
5. “Outsource to the institutional GME.”

| Table S1 | | ILS (%) | No ILS (%) | p-value |
| --- | --- | --- | --- | --- |
| “Patient safety and QI concepts are an important part of RO resident education” | | | | |
| Disagree | 0 | | 10.5 | 0.16 |
| Neutral | 5.8 | | 5.2 |  |
| Agree | 94.1 | | 84.2 |  |
| “Residents in my program are enthusiastic about patient safety and QI training” | | | | |
| Disagree | 5.9 | | 10.5 | 0.82 |
| Neutral | 17.6 | | 15.8 |  |
| Agree | 76.5 | | 47.3 |  |
| “Residents in my program are adequately exposed to patient safety and QI activities within my department” | | | | |
| Disagree | 12.8 | | 31.6 | 0.16 |
| Neutral | 17.6 | | 21.0 |  |
| Agree | 70.6 | | 47.4 |  |
| “Residents who graduate from my program are adequately prepared to meet the patient safety and QI expectations of clinical practice” | | | | |
| Disagree | 8.8 | | 15.8 | 0.32 |
| Neutral | 8.8 | | 21.0 |  |
| Agree | 79.4 | | 63.1 |  |

| Table S2 | ILS (%) | No ILS(%) | p-value |
| --- | --- | --- | --- |
| Resources Used In Creating and/or Improving Patient Safety and QI Training Programs | | | |
| Safety and QI Pubs | 55.9 | 47.4 | 0.28 |
| Curriculum Template | 41.2 | 36.8 | 0.54 |
| Online Module/Guide | 35.3 | 36.8 | 0.80 |
| M&M Guide | 29.4 | 31.6 | 0.78 |
| QI Leaders Outside Institution | 23.5 | 31.6 | 0.57 |
| Project Development Platform | 23.5 | 21.1 | 1.00 |
| Other | 14.7 | 10.5 | 0.67 |
| Curriculum Elements in Patient Safety and QI Training | | | |
| Clinical Safety/QI Activity | 67.6 | 68.4 | 0.53 |
| Didactic Activities | 67.6 | 57.9 | 0.49 |
| M&M Conference | 41.2 | 84.2 | 0.60 |
| Safety/QI Project Requirement | 41.2 | 36.8 | 0.64 |
| Patient Safety Rotation | 26.5 | 36.8 | 0.65 |
| Web-based Activities | 29.4 | 15.8 | 0.37 |
| Simulated Activities | 14.7 | 5.3 | 0.44 |
| Departmental Safety/QI Event | 14.7 | 36.8 | **0.05** |
| Other | 2.9 | 10.5 | 0.93 |
| Assessing Effectiveness of Safety/QI Training | | | |
| Resident Feedback | 64.7 | 47.4 | 0.23 |
| Faculty Feedback | 41.2 | 36.8 | 0.76 |
| Knowledge Assessment of Residents | 26.5 | 36.8 | 0.44 |
| Formal Evaluation of QI Project | 20.6 | 26.3 | 0.64 |
| Not Assessing | 20.6 | 15.8 | 0.64 |
| Patient Satisfaction | 5.9 | 21.1 | 0.08 |
| Other | 2.9 | 15.8 | 0.09 |
| Barriers Faced in Creating a Patient Safety/QI Training Program | | | |
| Lack of Resident Time | 44.0 | 25.8 | 0.65 |
| Lack of PD Time | 44.0 | 25.8 | 0.54 |
| Lack of PD expertise | 36.0 | 16.1 | 0.74 |
| Lack of Other Faculty Expertise | 32.0 | 9.7 | 0.59 |
| Other Activities are Sufficient | 32.0 | 22.6 | 0.42 |
| Lack of Funding | 24.0 | 19.4 | 0.21 |
| Lack of Interest By Residents | 20.0 | 22.6 | 0.07 |
| Lack of Safety/QI Activities | 16.0 | 6.5 | 1.00 |
| Other | 12.0 | 3.2 | 0.65 |
